# Greater addition of neurons to the olfactory bulb than to the cerebral cortex of eulipotyphlans but not rodents, afrotherians or primates

**DOI:** 10.3389/fnana.2014.00023

**Published:** 2014-04-11

**Authors:** Pedro F. M. Ribeiro, Paul R. Manger, Kenneth C. Catania, Jon H. Kaas, Suzana Herculano-Houzel

**Affiliations:** ^1^Instituto de Ciências Biomédicas, Universidade Federal do Rio de JaneiroRio de Janeiro, Brasil; ^2^Instituto Nacional de Neurociência Translacional, Ministério de Ciência e TecnologiaSão Paulo, Brasil; ^3^Department of Anatomy, University of the WitwatersrandJohannesburg, South Africa; ^4^Department of Biology, Vanderbilt UniversityNashville, TN, USA; ^5^Department of Psychology, Vanderbilt UniversityNashville, TN, USA

**Keywords:** olfactory bulb, cortical expansion, mosaic evolution, olfaction

## Abstract

The olfactory bulb is an evolutionarily old structure that antedates the appearance of a six-layered mammalian cerebral cortex. As such, the neuronal scaling rules that apply to scaling the mass of the olfactory bulb as a function of its number of neurons might be shared across mammalian groups, as we have found to be the case for the ensemble of non-cortical, non-cerebellar brain structures. Alternatively, the neuronal scaling rules that apply to the olfactory bulb might be distinct in those mammals that rely heavily on olfaction. The group previously referred to as Insectivora includes small mammals, some of which are now placed in Afrotheria, a base group in mammalian radiation, and others in Eulipotyphla, a group derived later, at the base of Laurasiatheria. Here we show that the neuronal scaling rules that apply to building the olfactory bulb differ across eulipotyphlans and other mammals such that eulipotyphlans have more neurons concentrated in an olfactory bulb of similar size than afrotherians, glires and primates. Most strikingly, while the cerebral cortex gains neurons at a faster pace than the olfactory bulb in glires, and afrotherians follow this trend, it is the olfactory bulb that gains neurons at a faster pace than the cerebral cortex in eulipotyphlans, which contradicts the common view that the cerebral cortex is the fastest expanding structure in brain evolution. Our findings emphasize the importance of not using brain structure size as a proxy for numbers of neurons across mammalian orders, and are consistent with the notion that different selective pressures have acted upon the olfactory system of eulipotyphlans, glires and primates, with eulipotyphlans relying more on olfaction for their behavior than glires and primates. Surprisingly, however, the neuronal scaling rules for primates predict that the human olfactory bulb has as many neurons as the larger eulipotyphlan olfactory bulbs, which questions the classification of humans as microsmatic.

## Introduction

The olfactory bulb is an evolutionarily ancient structure of the forebrain, found in nearly all vertebrates, including jawless fishes (Butler and Hodos, 2005). The olfactory bulb thus precedes the appearance of a well-developed mammalian cerebral cortex, and its expansion drove the initial increase in relative brain size in mammalian evolution (Rowe et al., 2011). However, the olfactory bulb has been shown to scale more slowly than the cerebral cortex in a number of modern mammalian species, although independently of the volume of the cerebral cortex across primates (Stephan and Andy, 1969). While the relative volume of the cerebral cortex when regressed onto body mass was once shown to increase with brain volume across living mammalian species, and to increase the most amongst primates (leading to the concept of cortical expansion in evolution; Stephan and Andy, 1969), the relative volume of the olfactory bulb decreases by the same measure in the species examined—but not in Insectivora (Stephan and Andy, 1969; Jolicoeur et al., 1984). This seemed consistent both with a basal position of Insectivora and with the progressive view of evolution still in vogue in the late 20th century.

At that time, Insectivora was a “scrap basket for small mammals” (Simpson, 1945), and viewed as an early offshoot in the placental mammal radiation, based on morphological criteria. In contrast, recent molecular data (Stanhope et al., 1998; Madsen et al., 2001; Murphy et al., 2001) provided convincing evidence that the group was polyphyletic, and actually consisted of two orders: afrosoricida (tenrecs and golden moles), under the superorder Afrotheria (Stanhope et al., 1998; Madsen et al., 2001; Murphy et al., 2001), and Eulipotyphla (hedgehogs, moles, shrews, solenodons; Waddell et al., 1999; Douady et al., 2002; Waddell and Shelley, 2003). Eulipotyphlans were later grouped into the Laurasiatheria clade, which includes bats, cetartiodactyls, carnivores, pangolins, and perissodactyls (Madsen et al., 2001; Murphy et al., 2001). Thus, in contrast from the original view that placed insectivores as a whole as an early branch at the root of placental mammals, this place is now occupied by Afrotherians (which do include golden moles, some of the original insectivores) and Xenarthra (Madsen et al., 2001; Murphy et al., 2001). The remainder of the original insectivores, now known to be eulipotyphlans, are considered a later offshoot, at the base of Laurasiatheria (Madsen et al., 2001; Murphy et al., 2001).

We have previously demonstrated that the scaling rules that relate the mass of the cerebral cortex and cerebellum to their numbers of neurons differ across primates, glires and eulipotyphlans (reviewed in Herculano-Houzel, 2011). While in primates these structures scale linearly in mass with their number of neurons (Herculano-Houzel et al., 2007), in glires (rodents and lagomorphs) they grow in mass with the number of neurons raised to the power of 1.7 and 1.2, respectively, which indicates that average neuronal size increases with number of neurons (Herculano-Houzel et al., 2006, 2011). In eulipotyphlans, the cerebral cortex scales in mass faster than it gains neurons, as it does in rodents, while the cerebellum increases in mass as a linear function of its numbers of neurons, as in primates (Sarko et al., 2009). These differences suggest that the cerebral cortex and cerebellum of primate, glires and eulipotyphlan brains are built according to cellular scaling rules that have diverged from their common ancestor. In contrast, the cellular scaling rules that apply to the remaining areas of the brain, excluding the olfactory bulb (that is, the ensemble of brainstem, diencephalon and basal ganglia), appear to be more similar across these three mammalian orders, and might possibly be shared among them (Sarko et al., 2009; Herculano-Houzel, 2011). This raises the possibility that the olfactory bulb, being a structure as evolutionarily ancient as the brainstem and antedating the expansion of the cerebral cortex, might also vary in mass according to neuronal scaling rules that are shared across mammalian orders.

Alternatively, the neuronal scaling rules that apply to the olfactory bulb may have been preserved in some mammalian orders, but not in others, in a pattern that might shed light on the history of brain evolution. For instance, if afrotherians are an early diverging group in mammalian evolution, any scaling rules that are shared by this group and others might be a characteristic of the common ancestor to eutherians. Here we investigate the rules that apply to the scaling of the mass of the olfactory bulb as a function of its number of neurons in glires, primates, and the two clades once lumped as Insectivora: afrotherians and eulipotyphlans, by using the isotropic fractionator (Herculano-Houzel and Lent, 2005) to determine the cellular composition of the olfactory bulb in 24 species, a method that was recently shown to give results that are comparable to those obtained with stereology (Bahney and von Bartheld, 2014). Further, we investigate how numbers of neurons in the olfactory bulb relate to numbers of neurons in the whole brain (without the olfactory bulb) and particularly in the cerebral cortex across species, to examine the notion that the cerebral cortex is the fastest-scaling structure in the brain and its corollary: that the decrease in relative size of the olfactory bulb is equivalent to a decrease in the relative number of neurons in the olfactory bulb as the brain gains neurons across species.

## Materials and methods

We applied the isotropic fractionator (Herculano-Houzel and Lent, 2005) to determine the cellular composition of the olfactory bulbs of 10 glire species and 6 primate and the closely related Scandentia species. Additionally, we compare our data to the cellular composition of the olfactory bulbs of 5 insectivore species (Sarko et al., 2009) and 3 afrotherian species (Neves et al., 2014), analyzed previously in the same manner.

### Animals

A total of 73 olfactory bulbs were collected from primates, rodents and one scandentia whose brains were prepared for the examination of cerebral structures in unrelated experiments. From primates, 25 olfactory bulbs were collected from 6 galagos (*Otolemur Garnettii*, 11 bulbs), 3 marmosets (*Callithrix jacchus*, 5 bulbs), 3 owl monkeys (*Aotus trivirgatus*, 6 bulbs), one rhesus monkey (*Macaca mulatta*, 1 bulb), and one mouse lemur (*Microcebus* sp., 2 bulbs). From Glires (rodents plus lagomorphs), we collected a total of 32 olfactory bulbs from 9 gray squirrels (*Sciureus carolinensis*, 9 bulbs), 5 Wistar rats (*Rattus norvegicus*, 5 bulbs), 2 agouti (*Dasyprocta primnolopha*, 2 bulbs), 2 swiss mice (*Mus musculus*, 4 bulbs), 2 golden hamsters (*Mesocrycetus auratus*, 2 bulbs), 2 guinea pigs (*Cavia porcellus*, 2 bulbs), 2 capybaras (*Hydrochoerus hydrochoeris*, 2 bulbs), 1 rabbit (*Oryctolagus cuniculus*, 1 bulb), 3 naked mole-rats (*Heterocephalus glaber*, 3 bulbs) and one Cayenne spiny rat (*Proechimys guyannensis*, 2 bulbs); some of the present data on olfactory bulbs were published previously in Herculano-Houzel et al. (2011). We also collected 16 olfactory bulbs from 10 tree shrews (*Tupaia glis*). All animals were adults at the time of the experiments. All non-human primates, except for the mouse lemur, were raised in a colony in the Department of Psychology at Vanderbilt University. The mouse lemur brain was acquired from the Duke University Lemur Center.

Insectivore data were obtained from Sarko et al. (2009) and consist of the species averages of 34 olfactory bulbs from 5 species: short-tailed shrew (*Blarina carolinensis*, 5 animals, 10 bulbs), star-nosed mole (*Condylura cristata*, 4 animals, 8 bulbs), smoky shrew (*Sorex fumeus*, 3 animals, 6 bulbs), hairy-tailed mole (*Parascalops breweri*, 3 animals, 6 bulbs) and eastern mole (*Scalopus aquaticus*, 3 animals, 6 bulbs). Afrotherian data were obtained from Neves et al. (2014) and consist of species averages of 5 olfactory bulbs from 3 species: elephant shrew (*Elephantulus myurus*, 2 animals, 2 bulbs), four-toed elephant shrew (*Petrodomus tetradactylus*, 2 animals, 2 bulbs), and rock hyrax (*Procavia capensis*, 1 animal, 1 bulb).

### Tissue

Olfactory bulbs were processed separately for each hemisphere; in most cases, both olfactory bulbs were available. Since no significant differences were observed between left and right olfactory bulb mass or number of neurons (2-tailed Student *t*-test, *p* > 0.05), all data were pooled together and presented as average values for both left and right olfactory bulbs for each species.

Numbers of neurons in other brain structures of the respective species were obtained from our previous studies using the same counting methodology (Herculano-Houzel et al., 2006, 2007; Sarko et al., 2009; Gabi et al., 2010; Neves et al., 2014). All numbers presented here refer to both olfactory bulbs on the two sides of the brain, and to the whole brain, cerebral cortex or cerebellum, as described in our previous studies (Herculano-Houzel et al., 2006, 2007, 2011; Sarko et al., 2009; Gabi et al., 2010).

### Dissection

All primates and scandentia, except for the mouse lemur, were sacrificed by lethal injection of sodium pentobarbital, and perfused transcardially with 0.9% phosphate-buffered saline followed by 4% phosphate-buffered paraformaldehyde. Rodents were sacrificed by inhalation of ether and perfused transcardially as above. Brains were removed from the skull with care not to damage the olfactory bulbs, which were then removed by transecting the olfactory tract immediately proximal to the bulb. Due to variation across species, this procedure may include unknown amounts of the anterior olfactory nucleus in the olfactory bulb. However, for the sake of consistency, all care was taken to ensure that the transaction of the olfactory tract was performed at a level where the olfactory tract was completely exposed, leaving all tissue surrounded by gray matter included in the structure that we refer to as the olfactory bulb. The olfactory bulbs were then weighed, and post-fixed for 2 weeks to 12 months by immersion in 4% phosphate-buffered paraformaldehyde.

### Isotropic fractionator

Total numbers of cells, neurons, and non-neuronal cells were estimated as described previously using the Isotropic Fractionator method (Herculano-Houzel and Lent, 2005). Briefly, each olfactory bulb is turned into an isotropic suspension of isolated nuclei of known, defined volume, kept homogeneous by agitation. The total number of nuclei in suspension—and therefore the total number of cells in the original tissue—is estimated by determining the density of nuclei in small aliquots stained with the fluorescent DNA marker DAPI (4′-6-diamidino-2-phenylindole dihydrochloride; Invitrogen), under the microscope. Once the total cell number is known, the proportion of neurons is determined by immunocytochemical detection of Neuronal Nuclear antigen (NeuN, mab377, Millipore), expressed in all nuclei of most neuronal cell types and not in non-neuronal cells (Mullen et al., 1992). Estimates of the proportion of NeuN-positive nuclei are considered reliable since the coefficient of variation among animals of the same species is typically well below 0.15. Numbers of non-neuronal cells are derived by subtraction.

Mitral cells in the olfactory bulb constitute the main output of the olfactory bulb, along with tufted cells, and are one of the neuronal subtypes known not to express NeuN (Mullen et al., 1992). Mitral cell neurons are therefore included in the NeuN-negative (other, non-neuronal cells) population in our study, and thus artificially inflate the count of non-neuronal cells in the olfactory bulb, while tufted cells are supposedly included as NeuN-positive neurons. However, mitral cells are vastly outnumbered by granule cell neurons and other cells in the olfactory bulb. In the mouse, the mitral cell layer (which includes other, GABAergic cell types) represents only 7.9% of all neurons in the olfactory bulb of the mouse, and tufted cells are estimated at 3–6% of all neurons (Parrish-Aungst et al., 2007). While it will definitely be important to quantify mitral and tufted cells separately, we could not at present quantify them. We focused, instead, on total numbers of neurons in the olfactory bulb, which are mostly located in the granule cell layer and in the glomerular layer. In the mouse main olfactory bulb, for instance, 52.6 and 34.0% of all neurons are found in the glomerular and granule cell layers, respectively (Parrish-Aungst et al., 2007). Given that non-neuronal cells are typically at least half of all cells in the olfactory bulb, we consider that the misplacement of mitral cell neurons as non-neuronal cells does not significantly influence the estimates of total numbers of neurons or non-neuronal cells in the main olfactory bulb reported here.

### Data analysis

All statistical analyses and regressions were performed in JMP 9.0 (SAS, USA), using the average values obtained from the individuals of each species. Correlations between variables were calculated using the Spearman correlation coefficient. If a significance criterion of *p* < 0.05 was reached, regressions of the data to linear and power functions were calculated. Exponents are presented ± standard error. In all analyses, “whole brain” refers to the sum of cerebral cortex, cerebellum and remaining areas, not including the olfactory bulb, as in our previous studies (Herculano-Houzel et al., 2007, 2011; Sarko et al., 2009; Gabi et al., 2010). Analysis of numbers of cells exclude the capybara, for which the quality of the preparation was doubtful, yielding numbers of cells that were less than half the expected.

## Results

**Figure 1:**
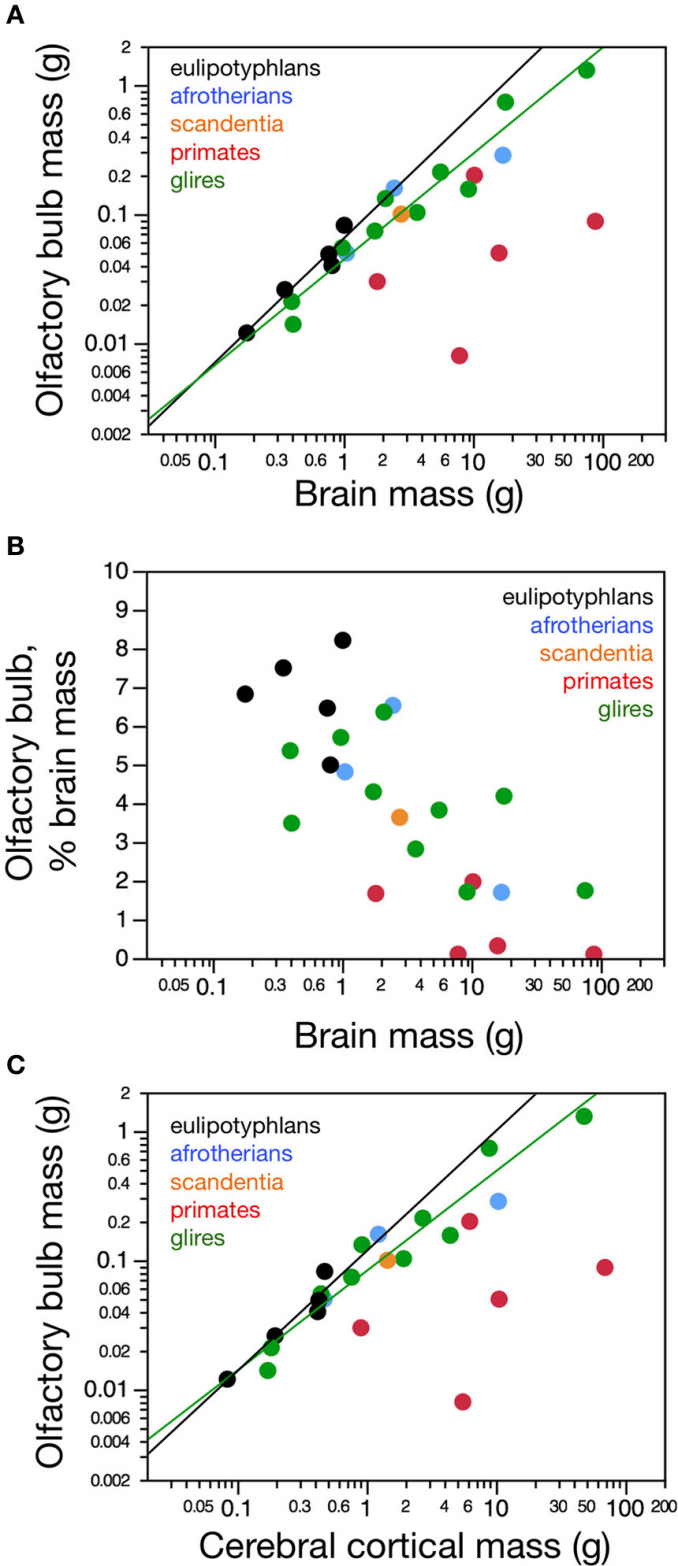
**Distribution of absolute and relative olfactory bulb mass across species**. **(A)** Average mass of both olfactory bulbs in each species and **(B)** relative mass of the olfactory bulbs expressed as a percentage of brain mass, both shown as a function of absolute brain mass (which does not include the olfactory bulbs). Olfactory bulb mass varies linearly with brain mass in eulipotyphlans (**A**, black; power function exponent, 0.965 ± 0.149, *p* = 0.0075), such that its relative size does not vary systematically with brain mass (**B**, black). In glires, olfactory bulb mass increases more slowly than brain mass (**A**, green; power function exponent, 0.820 ± 0.074, *p* < 0.0001), such that the relative mass of the olfactory bulb decreases with increasing brain mass (**B**, green). Afrotherians (blue) overlap with both eulipotyphlans and glires. In primates and scandentia there is no significant correlation between olfactory bulb mass and brain mass (**A** and **B**, red and orange). **(C)** The mass of the olfactory bulb increases linearly with the mass of the cerebral cortex across eulipotyphlans (black; exponent, 0.930 ± 0.170, *p* = 0.0120) and sublinearly across glires (green; exponent, 0.772 ± 0.073, *p* < 0.0001), but there is no significant correlation between olfactory bulb mass and cortical mass across primates (red). Eulipotyphlan data from Sarko et al. (2009). Afrotherian data from Neves et al. (2014). Glires, data from Herculano-Houzel et al. (2011) and this study. Primates and scandentia, data from this study.

Olfactory bulb size varies greatly within and between mammalian orders. In our sample of 24 species of glires, eulipotyphlans, afrotherians, primates, and scandentia, we find a 162.8-fold variation between the smallest and the largest olfactory bulb (0.008 g in the marmoset against 1.302 g in the capybara). Within orders, olfactory bulb size varies 25-fold in primates (from 0.008 g in the marmoset to 0.200 g in the galago), 93-fold in glires (from 0.014 g in the mouse to 1.302 g in the capybara), and 7x in eulipotyphlans (from 0.012 g in the smoky shrew to 0.082 g in the eastern mole; Table 1). The range of variation in olfactory bulb size is however overlapping across the orders, and 13 out of 21 species have olfactory bulbs within the range of 0.01–0.1 g (Figure 1A). The two largest olfactory bulbs belong to rodents (agouti and capybara), which are remarkably large compared to the other bulbs analyzed.

**Table 1:** **Cellular composition of the olfactory bulb**.

**Species**	**Mass, g**	**Neurons**	**% Neurons**	**Neurons/mg**
**GLIRES**				
*Mus musculus*	0.014 ± 0.004	3.89 × 10^6^ ± 1.25 ± 10^6^	41.1 ± 4.3	257,475 ± 34,036
*Mesocricetus auratus*	0.055 ± 0.011	5.75 × 10^6^ ± 0.35 ± 10^6^	52.1 ± 12.0	105,418 ± 27,498
*Rattus norvegicus*	0.074 ± 0.022	11.10 × 10^6^ ± 3.20 ± 10^6^	54.4 ± 4.1	152,373 ± 26,913
*Proechimys cayennensis*	0.132	9.14 × 10^6^	30.2	69,254
*Cavia porcellus*	0.103 ± 0.013	6.06 × 10^6^ ± 1.30 ± 10^6^	38.2 ± 5.0	58,560 ± 5,340
*Sciurus carolinensis*	0.212 ± 0.022	28.84 × 10^6^ ± 7.90 ± 10^6^	43.4 ± 12.1	137,532 ± 38,236
*Oryctolagus cuniculus*	0.156	18.76 × 10^6^	45.0	120,288
*Dasyprocta primnolopha*	0.737 ± 0.162	58.12 × 10^6^ ± 4.95 ± 10^6^	44.9 ± 4.7	88,008 ± 14,973
*Hydrochoerus hydrochoeris*	1.302 ± 0.031	28.56 × 10^6^ ± 8.52 ± 10^6^	30.0 ± 1.6	21,864 ± 6018
**PRIMATES**				
*Microcebus murinus*	0.030 ± 0.008	7.64 × 10^6^ ± 0.12 ± 10^6^	44.0 ± 1.8	270,894 ± 61,946
*Callithrix jacchus*	0.008 ± 0.014	2.11 × 10^6^ ± 0.98 ± 10^6^	45.2 ± 7.2	232,309 ± 137,605
*Otolemur garnetti*	0.200 ± 0.016	30.24 × 10^6^ ± 9.64 ± 10^6^	46.3 ± 7.3	149,219 ± 43,590
*Aotus trivirgatus*	0.050 ± 0.012	7.92 × 10^6^ ± 3.11 ± 10^6^	48.4 ± 11.5	155,879 ± 62,241
*Macaca mulatta*	0.088	8.47 × 10^6^	43.5	96,293
**SCANDENTIA**				
*Tupaia glis*	0.100 ± 0.032	12.7 × 10^6^ ± 3.58 ± 10^6^	38.3 ± 6.0	130,173 ± 17,451
**EULIPOTYPHLA**				
*Sorex fumeus*	0.012 ± 0.002	3.33 × 10^6^ ± 1.05 ± 10^6^	53.8 ± 6.4	289,806 ± 124,350
*Blarina brevicauda*	0.026 ± 0.003	8.09 × 10^6^ ± 0.94 ± 10^6^	62.2 ± 4.5	318,164 ± 34,950
*Parascalops breweri*	0.049 ± 0.008	16.75 × 10^6^ ± 6.37 ± 10^6^	60.5 ± 5.6	333,590 ± 81,590
*Condylura cristata*	0.040 ± 0.005	10.55 × 10^6^ ± 4.29 ± 10^6^	57.1 ± 9.2	254,720 ± 74,620
*Scalopus aquaticus*	0.082 ± 0.005	34.61 × 10^6^ ± 5.96 ± 10^6^	65.8 ± 6.5	423,520 ± 94,950
**AFROTHERIA**				
*Petrodomus tetradactylus*	0.159 ± 0.013	12.83 × 10^6^ ± 0.54 ± 10^6^	48.2 ± 11.8	80,805 ± 3084
*Elephantulus myurus*	0.050 ± 0.014	9.69 × 10^6^ ± 2.47 ± 10^6^	66.5 ± 1.5	194,678 ± 5708
*Procavia capensis*	0.286	20.91 × 10^6^	58.6	73,110

All values refer to both olfactory bulbs. % Neurons, percentage of cells in the olfactory bulb that are NeuN+. Neurons/mg, density of neurons per miligram of olfactory bulb tissue. Data from Sarko et al. (2009), Herculano-Houzel et al. (2011), Neves et al. (2014) and this study.

### Olfactory bulb mass

Across species, larger glire and insectivore brains (excluding the olfactory bulb) tend to have larger olfactory bulbs, with overlapping distributions of brain and olfactory bulb mass that also include afrotherians (Figure 1A). Olfactory bulb mass in insectivores varies as a power function of brain mass (which doesn't include the olfactory bulb) with exponent 0.965 ± 0.149 (*r*^2^ = 0.933, *p* = 0.0075), which is indistinguishable from unity (Figure 1A). Indeed, the relative mass of the eulipotyphlan olfactory bulb in proportion to the brain does not vary systematically with increasing brain mass (Spearmann correlation, ρ = 0.100, *p* = 0.8729; Figure 1B), and ranges between 4.99 and 8.21%. In glires, olfactory bulb mass varies as a power function of brain mass of exponent 0.820 ± 0.074 (*p* < 0.0001), which is significantly different from unity. As a consequence, the relative mass of the olfactory bulbs in proportion to the brain (average of 3.94 ± 0.50%) decreases with increasing brain mass in glires (exponent −0.180 ± 0.0074, *p* = 0.0409), varying from 1.71% (in the rabbit) to 6.35% of brain mass (in the spiny rat; Figure 1B). Afrotherians overlap with both glires, with an average relative mass of the olfactory bulbs of 4.34 ± 1.41% of brain mass. Across primate and scandentia species, in turn, there is no significant correlation between olfactory bulb mass and brain mass (Spearmann correlation, ρ = 0.429, *p* = 0.3374; Figure 1A). Thus, while larger brains are accompanied by larger olfactory bulbs across both eulipotyphlan and glire species, the relative size of the olfactory bulbs compared to the brain does neither increase nor decrease with brain size in insectivores or primates, but it decreases with increasing brain size in glires. However, there is a significant negative correlation between the relative mass of the olfactory bulb and brain mass across all orders combined (Spearman correlation, ρ = −0.736, *p* < 0.0001; Figure 1B). The relative mass of the olfactory bulb is smaller in primates (0.01–3.63% of brain mass) than in glires, and smaller in glires than in eulipotyphlans, for a comparable range of brain mass (Figure 1B).

The mass of the olfactory bulb varies linearly with the mass of the cerebral cortex across eulipotyphlans (power exponent, 0.930 ± 0.170, *p* = 0.0120), and slightly below linearity across glires (power exponent, 0.772 ± 0.073, *p* < 0.0001). Afrotherians overlap with both eulipotyphlans and glires. There is no significant correlation between olfactory bulb mass and cortical mass across primates (Figure 1C).

### Cellular scaling rules for the olfactory bulb

**Figure 2:**
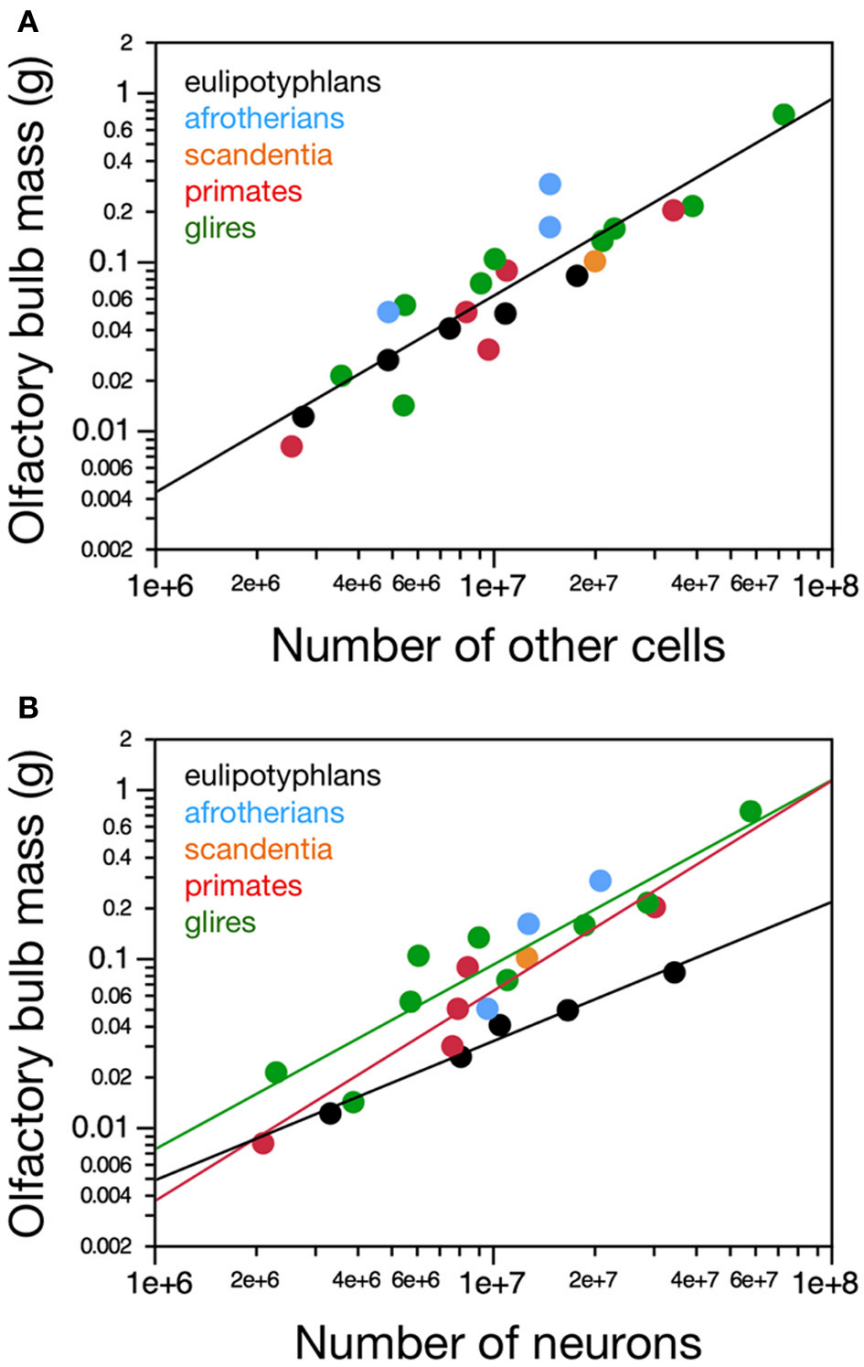
**Neuronal and non-neuronal scaling rules for the olfactory bulb**. Graphs show the relationship between average mass of both olfactory bulbs in each species and **(A)** the total number of other (non-neuronal) cells or **(B)** the total number of neurons in them. **(A)** Olfactory bulb mass varies similarly, and linearly, with its number of other cells across the ensemble of eulipotyphlans (black), glires (green), primates, and scandentia (red and orange), but **(B)** as a power function of its number of neurons with an exponent of 0.823 ± 0.071 in eulipotyphlans, 1.092 ± 0.170 in glires, and 1.243 ± 0.192 in primates and scandentia. Notice that eulipotyphlans have more neurons packed into olfactory bulbs of a similar mass in glires or afrotherians. Eulipotyphlan data from Sarko et al. (2009). Afrotherian data from Neves et al. (2014). Glires, data from Herculano-Houzel et al. (2011) and this study. Primates and scandentia, data from this study.

Despite the different absolute and relative sizes of the olfactory bulb across species and orders, the relationship between olfactory bulb mass and its number of non-neuronal cells is overlapping among eulipotyphlans, glires, afrotherians and primates (Figure 2A). For the ensemble of species, olfactory bulb mass can be expressed equally well as a linear function of its numbers of non-neuronal cells (*r*^2^ = 0.847, *p* < 0.0001) and as a power function of the number of these cells with exponent 1.164 ± 0.115, which includes unity (*p* < 0.0001; Figure 2A). This indicates that the olfactory bulbs of eulipotyphlans, glires, afrotherians, primates, and scandentia share non-neuronal scaling rules, which agrees with previous observations for the cerebral cortex, cerebellum and remaining brain areas across mammalian orders (Herculano-Houzel, 2011).

In contrast, different sets of neuronal scaling rules apply to the olfactory bulb of primates, glires and eulipotyphlans. Variations in olfactory bulb mass are best described as a linear function of the number of neurons in the structure both in primates (*r*^2^ = 0.929, *p* = 0.0020, against power exponent 1.243 ± 0.192, *r*^2^ = 0.912, *p* = 0.0030) and in glires (*r*^2^ = 0.937, *p* = 0.0002, against power exponent *r*^2^ = 0.856, 1.092 ± 0.170, *p* = 0.0004; Figure 2B.). Across eulipotyphlans, however, olfactory bulb mass is best described as a power law of the number of neurons with a smaller exponent of 0.823 ± 0.071, which excludes linearity (*r*^2^ = 0.978, *p* < 0.0014; Figure 2B). Afrotherians overlap with the relationship for glires, not eulipotyphlans (Figure 2B, blue). Combined with the shared, near-linear scaling of olfactory bulb mass with numbers of non-neuronal cells, these exponents suggest that average neuronal size in the olfactory bulb remains constant with bulb size across primate and glires species, but decreases as olfactory bulb mass increases in eulipotyphlans (Sarko et al., 2009). The direct comparison across species shows that olfactory bulbs of a similar mass above about 0.02 g are built with larger numbers of neurons in insectivores than in primates, glires or afrotherians—that is, similar numbers of neurons are packed into smaller olfactory bulbs in eulipotyphlans compared to primates, glires and afrotherians (Figure 2B).

**Figure 3:**
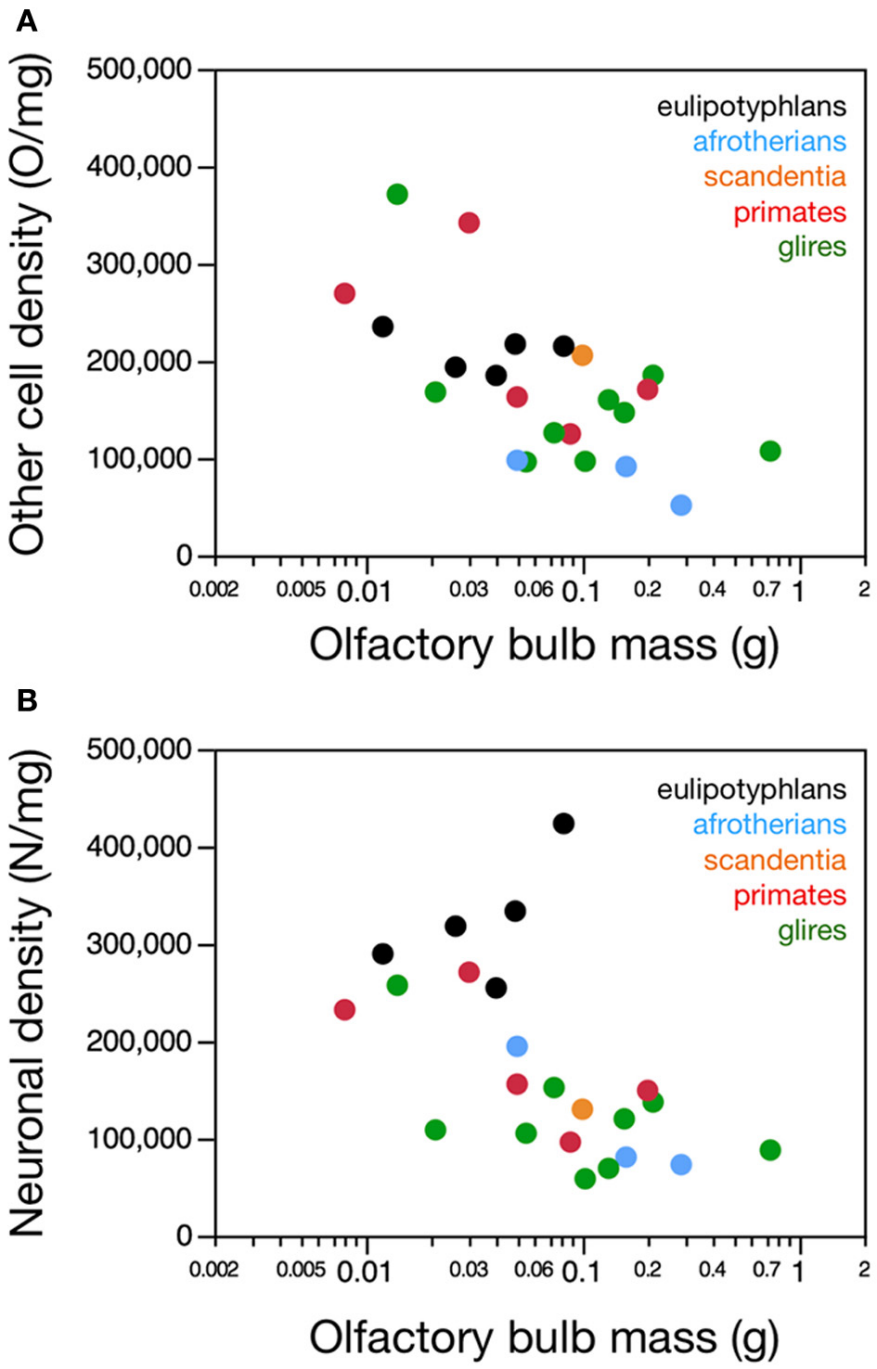
**Neuronal and non-neuronal cell densities in the olfactory bulb**. Graphs show the relationship between olfactory bulb mass and **(A)** average non-neuronal (other) cell density or **(B)** average neuronal density in the olfactory bulb across species. **(A)** There is no significant correlation between other cell density (in other cells/mg) and olfactory bulb mass across species within any order. **(B)** Neuronal cell density does not correlate with olfactory bulb mass in primates (and scandentia) or glires, but it does increase with larger olfactory bulb mass in eulipotyphlans when the outlier, the star-nosed mole, is excluded. Notice that neuronal densities in the olfactory bulb in eulipotyphlan species are higher than in most other species. Eulipotyphlan data from Sarko et al. (2009). Afrotherian data from Neves et al. (2014). Glires, data from Herculano-Houzel et al. (2011) and this study. Primates and scandentia, data from this study.

In agreement with the shared and linear non-neuronal scaling rules among orders, we find overlapping non-neuronal cell densities across most of the species that do not vary in correlation with bulb mass (Spearman correlation, all *p* > 0.3; Figure 3A). In contrast, eulipotyphlans exhibit much larger neuronal densities in the olfactory bulb than afrotherians, most glires, or primates (Figure 3B). In line with a linear relationship between bulb mass and number of neurons across glires and primates, we find no significant correlation between neuronal density and olfactory bulb mass in either group (Spearman correlation, *p* > 0.1; Figure 3B). In eulipotyphlans, as described before (Sarko et al., 2009), neuronal density does not vary significantly with bulb mass across the five species (Spearman correlation, ρ = 0.700, *p* = 0.1881). However, the star-nosed mole is an outlier in the group, with a much smaller neuronal density than would be expected based on the other four insectivore species examined (Sarko et al., 2009). If the star-nosed mole species is removed from the comparison, we find that neuronal density in the olfactory bulb increases significantly with bulb mass (ρ = 1.000, *p* = < 0.0001). As mentioned above, these data suggest that average neuronal size in the olfactory bulb does not vary systematically with increasing bulb size in primates of glires, but decreases in eulipotyphlans.

Consistently with the larger neuronal densities in insectivores than in the other two groups, eulipotyphlans also differ from the other orders in the percentage of olfactory bulb cells that are neurons. This percentage is higher in eulipotyphlans (59.9 ± 2.1%) than in afrotherians (48.0 ± 10.8%), primates and scandentia (44.4 ± 1.3%) or glires (43.2 ± 2.4%; ANOVA, *p* = 0.0101).

### Relative distribution of neurons in the olfactory bulb compared to the whole brain

**Figure 4:**
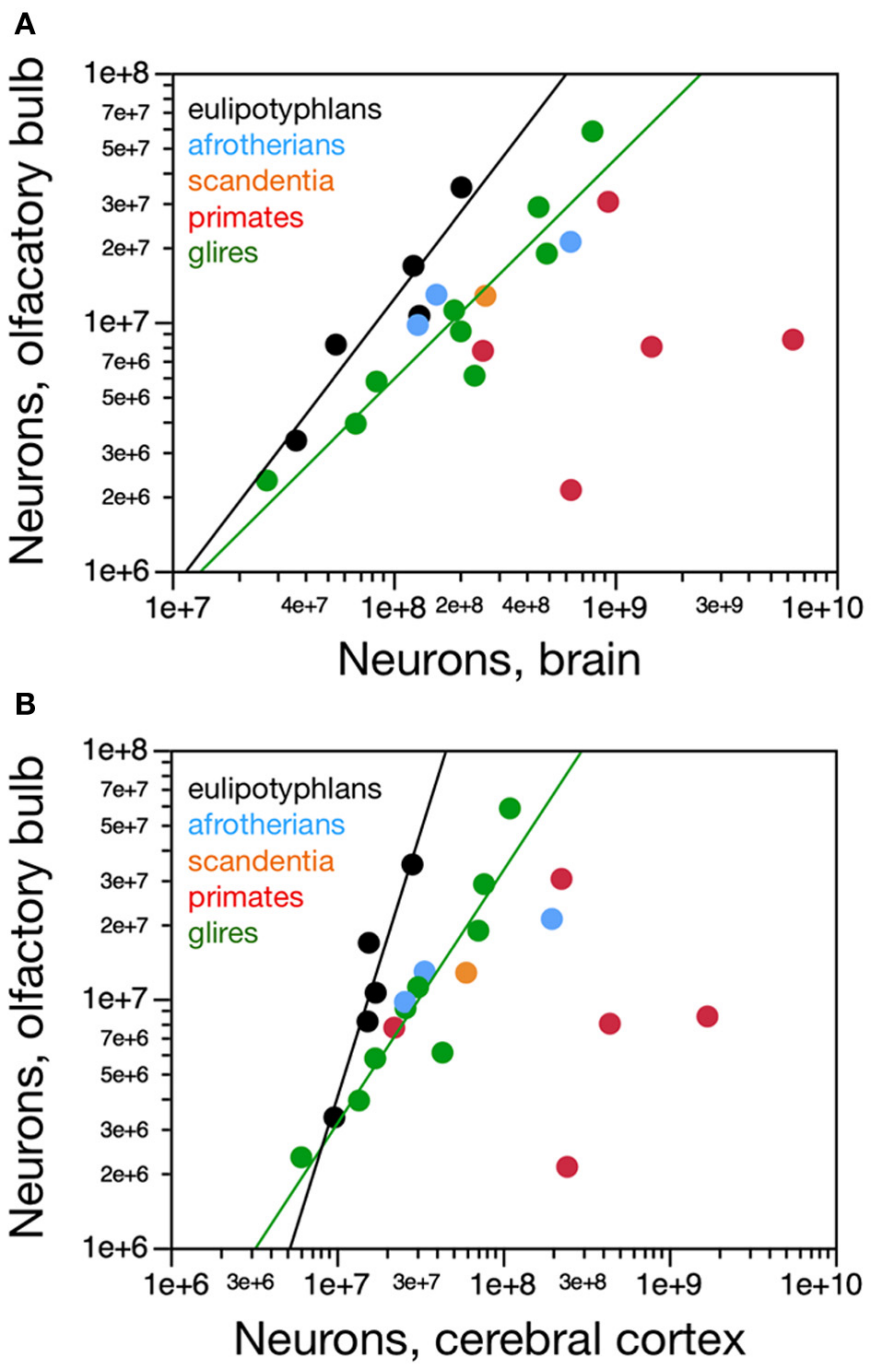
**Scaling of numbers of neurons in the olfactory bulb as a function of numbers of neurons in the brain and in the cerebral cortex**. **(A)** The number of neurons in the olfactory bulb increases as power functions of the number of neurons in the whole brain (without the olfactory bulb) of exponent 1.163 ± 0.247 in eulipotyphlans and 0.884 ± 0.122 in glires, respectively, but not in primates. Notice that eulipotyphlans have more neurons in the olfactory bulb than glires and afrotherians for similar numbers of brain neurons. **(B)** The number of neurons in the olfactory bulb of eulipotyphlans increases at a faster rate than in the cerebral cortex, as a power function of exponent 2.129 ± 0.428, but at the same pace as the cerebral cortex in glires, with an exponent of 1.018 ± 0.148. Notice that eulipotyphlans have more neurons in the olfactory bulb than glires and afrotherians for similar numbers of cortical neurons. There is no significant relationship between numbers of olfactory bulb and cerebral cortical neurons across primate species. Eulipotyphlan data from Sarko et al. (2009). Afrotherian data from Neves et al. (2014). Glires, data from Herculano-Houzel et al. (2011) and this study. Primates and scandentia, data from this study.

Just as larger glire and eulipotyphlan (but not primate) brains accompany larger olfactory bulbs (Figure 1), larger numbers of neurons in the brain are accompanied by larger numbers of neurons in the olfactory bulb in eulipotyphlans and glires, but not in primates (Spearman correlation, ρ = 0.900, 0.891, and 0.257, *p* = 0.0374, 0.0005, and 0.6228, respectively; Figure 4A). In both eulipotyphlans and glires, the rate of addition of neurons to the olfactory bulb as a function of number of neurons in the whole brain is indistinguishable from linearity (power exponents, 1.163 ± 0.247 and 0.884 ± 0.122, *r*^2^ = 0.881 and 0.883, *p* = 0.0182 and 0.0002, respectively; linear fit, *r*^2^ = 0.856 and 0.896, *p* = 0.0243 and 0.0001; Figure 4A).

**Figure 5:**
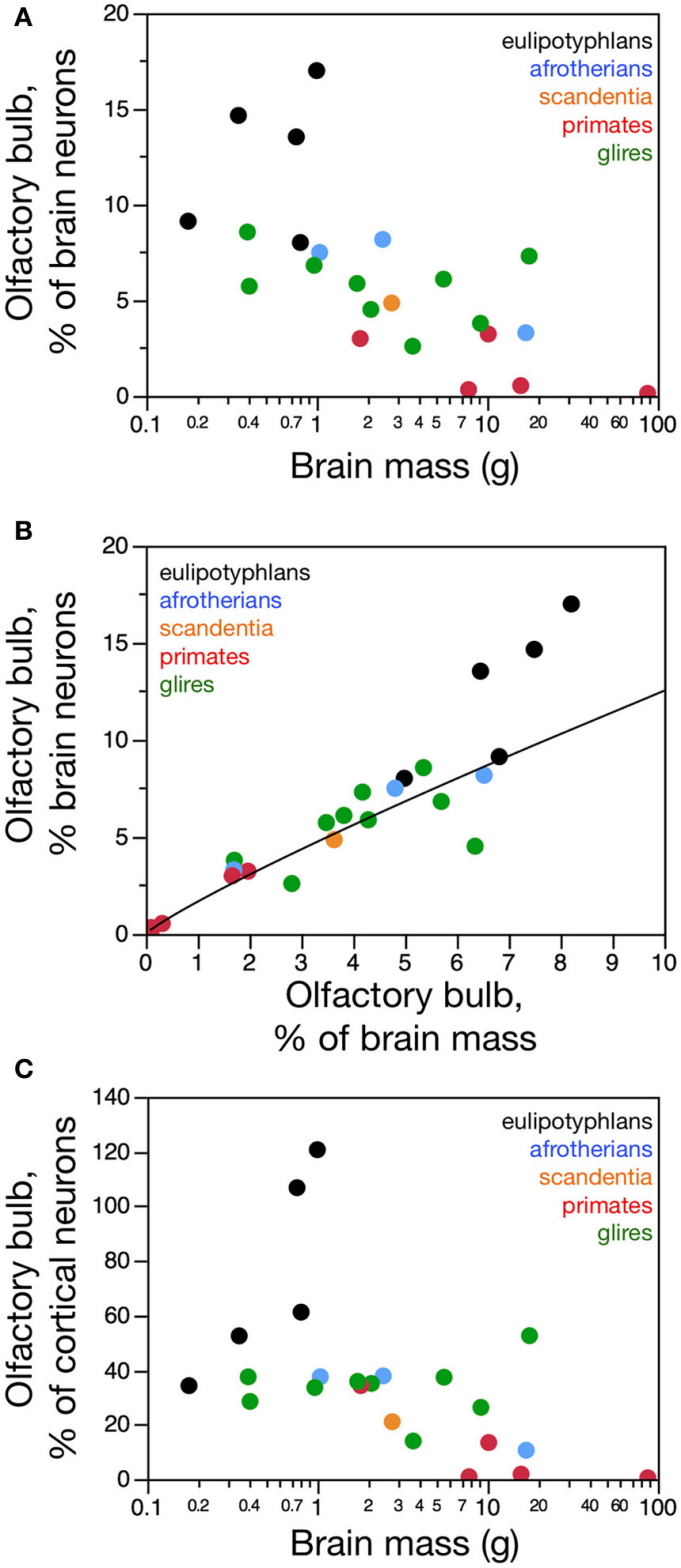
**Scaling of relative numbers of neurons in the olfactory bulb compared to the brain and cerebral cortex**. **(A)** The relative number of neurons in the olfactory bulb expressed as a percentage of the number of neurons in the brain (excluding the olfactory bulb) is not significantly correlated with brain mass within any of the mammalian orders examined, although it decreases with increasing brain mass across all species combined. **(B)** The relative number of neurons in the olfactory bulb increases with increasing relative mass of the olfactory bulb (both compared to the whole brain) across primates, glires and afrotherians combined, as a power function of exponent 0.877 ± 0.054. **(C)** The relative number of neurons in the olfactory bulb expressed as a percentage of the number of neurons in the cerebral cortex is not significantly correlated with brain mass within any of the mammalian orders examined. Eulipotyphlan data from Sarko et al. (2009). Afrotherian data from Neves et al. (2014). Glires, data from Herculano-Houzel et al. (2011) and this study. Primates and scandentia, data from this study.

Eulipotyphlans, however, have more neurons in the olfactory bulb than glires and afrotherians for a similar number of brain neurons (Figure 4A). Indeed, the eulipotyphlan olfactory bulb has on average 12.5 ± 1.7% of the total number of neurons in the brain (which does not include the olfactory bulb), while in afrotherians the olfactory bulb has 6.3 ± 1.5% of the neurons in the brain, in glires it has 5.3 ± 0.7% of the number found in the brain, and in primates, only 2.0 ± 0.8% (ANOVA, *p* < 0.0001; Figure 5A). As expected from the linear relationships between numbers of neurons in the olfactory bulb and in the brain, in none of the orders is the relative number of neurons in the olfactory bulb expressed as a percentage of the number of brain neurons significantly correlated with brain mass (Spearman correlations: insectivores, *p* = 0.6238; glires, 0.1739; primates and scandentia, 0.2080; Figure 5A). Thus, numbers of neurons increase concertedly between the olfactory bulb and the brain in eulipotyphlans and glires (while the former have larger absolute and relative numbers of neurons in the olfactory bulb than the latter for a same number of brain neurons), but are independent of one another in primates. However, there is a significant negative correlation between the relative number of neurons in the olfactory bulb and brain mass across all orders (Spearman correlation, ρ = −0.728, *p* < 0.0001; Figure 5A). Because the relative mass of the olfactory bulb is also negatively correlated with brain mass across all orders (Figure 1B), the relationship between relative number of neurons and relative mass of the olfactory bulb (both expressed as percentages of whole brain) can be described as a single power function across primates, glires and scandentia, with an exponent that approaches linearity (0.877 ± 0.054, *r*^2^ = 0.942, *p* < 0.0001), although 3 species of Eulipotyphla remain outliers in the relationship (Figure 5B).

Although the eulipotyphlan olfactory bulb gains neurons linearly with the brain as a whole, it gains neurons at a faster rate than the cerebral cortex, as a power function of the number of cortical neurons of exponent 2.129 ± 0.428, which excludes unity and, therefore, linearity (Figure 4B). In eulipotyphlans, the number of neurons in the olfactory bulb represents on average 75.0 ± 16.5% of the number of neurons in the cerebral cortex, and actually exceeds the latter in two species (eastern mole and hairy-tailed mole, the two with the largest olfactory bulbs amongst insectivores in the sample; Figure 5C). As expected from the supralinear rate of addition of neurons to the olfactory bulb compared to the cerebral cortex, the relative number of neurons in the former structure expressed as a percentage of the latter increases significantly with brain mass across eulipotyphlans (Spearman correlation, ρ = 0.900, *p* = 0.0374). In constrast, the olfactory bulb in glires gains neurons at the same pace as the cerebral cortex (power exponent, 1.018 ± 0.148, *r*^2^ = 0.872, *p* = 0.0002; linear fit, *r*^2^ = 0.862, *p* = 0.0003; Figure 4B), and represents on average only 31.0 ± 3.9% of the neurons found in the cerebral cortex, a percentage that does not vary significantly together with brain mass in glires (Spearman correlation, *p* = 0.4888). Similarly, the afrotherians examined have a number of neurons in the olfactory bulb that represents 28.6 ± 9.0% of the number of neurons found in the cerebral cortex (Figure 5C). Eulipotyphlans, in comparison, have more neurons in the olfactory bulb than glires and afrotherians for a similar number of neurons in the cerebral cortex (Figure 4B). In contrast, in primates and scandentia, the number of neurons in the olfactory bulb represents only 12.0 ± 5.6% of the number of neurons in the cerebral cortex, and there is no significant correlation between numbers of neurons across the two structures (Spearman correlation, *p* = 0.2080; Figure 4B).

**Figure 6:**
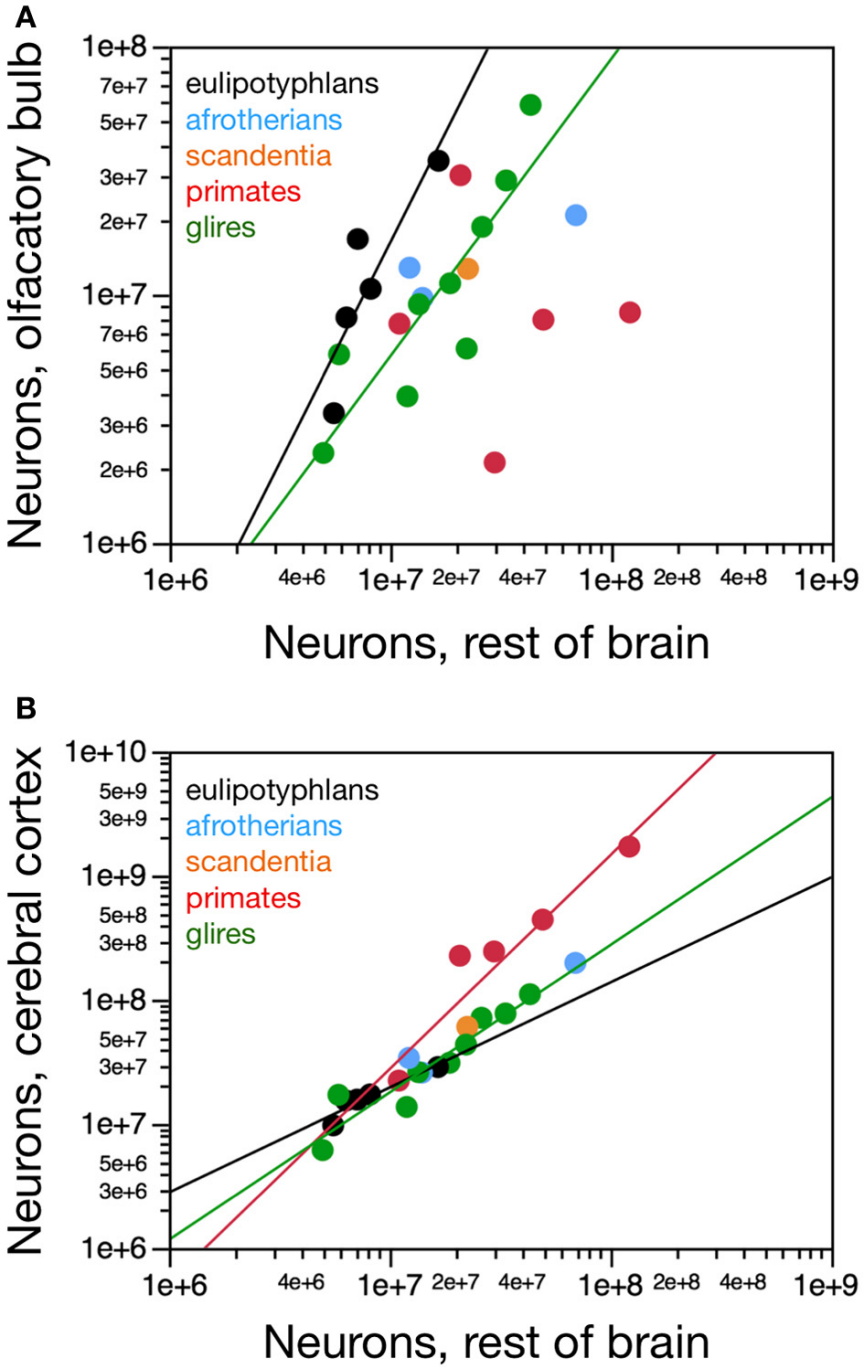
**Scaling of numbers of neurons in the olfactory bulb and in the cerebral cortex as a function of numbers of neurons in the rest of brain**. **(A)** The number of neurons in the olfactory bulb increases as power functions of the number of neurons in the rest of brain of exponent 1.770 ± 0.578 in eulipotyphlans and 1.198 ± 0.252 in glires, respectively, but not in primates. Notice that eulipotyphlans have more neurons in the olfactory bulb than glires and afrotherians for similar numbers of rest of brain neurons. **(B)** The number of neurons in the cerebral cortex of eulipotyphlans increases more slowly than the number of neurons in the rest of brain, as a power function of exponent 0.846 ± 0.180, but at the same pace as the cerebral cortex in glires, with an exponent of 1.018 ± 0.148. Notice that eulipotyphlans have more neurons in the olfactory bulb than glires and afrotherians for similar numbers of cortical neurons. There is no significant relationship between numbers of olfactory bulb and cerebral cortical neurons across primate species. Eulipotyphlan data from Sarko et al. (2009). Afrotherian data from Neves et al. (2014). Glires, data from Herculano-Houzel et al. (2011) and this study. Primates and scandentia, data from this study.

**Figure 7:**
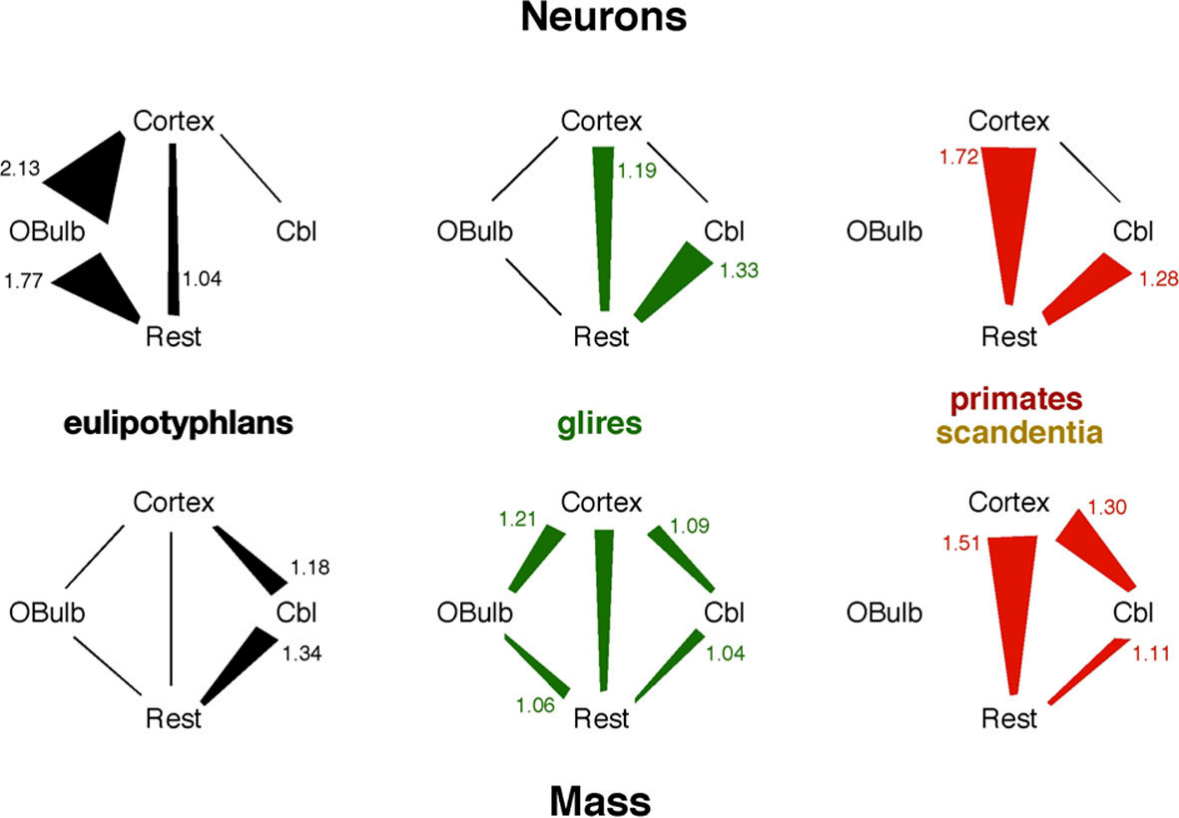
**Scaling relationships for neurons and mass across structures in each mammalian order**. Lines indicate linear scaling in numbers of neurons (top) or mass (bottom) across structures; polygons represent reciprocal power laws in the scaling of one structure against another, where the power law with the exponent larger than 1 is the larger side of the polygon and has its exponent indicated in the figure. Notice that the faster scaling of number of neurons in the eulipotyphlan olfactory bulb than in the cerebral cortex would not be predicted from the mass relationships across the two structures.

The eulipotyphlan olfactory bulb gains neurons at a faster rate than the non-cortical, non-cerebellar areas of the brain (“rest of brain”), as a power function of the number of rest of brain neurons of exponent 1.770 ± 0.578 (*r*^2^ = 0.758, *p* = 0.0548; Figure 6A) or as a linear function (*r*^2^ = 0.906, *p* = 0.0127). The olfactory bulb also has more neurons in eulipotyphlans than in glires, afrotherians or primates with a similar number of neurons in the rest of brain (Figure 6A). In eulipotyphlans, the cerebral cortex gains neurons more slowly than the rest of brain (power exponent, 0.846 ± 0.180, *r*^2^ = 0.880, *p* = 0.0182; Figure 6B), although this relationship can also be described as a linear function (*r*^2^ = 0.936, *p* = 0.0069). Despite the statistical uncertainty, the most likely scenario that best accounts for the scaling of numbers of neurons in the olfactory bulb in eulipotyphlans is that it gains neurons faster than the rest of the brain, while both structures gain neurons faster than the cerebral cortex, such that the olfactory bulb gains neurons even faster compared to the cortex than to the rest of brain. In glires, the olfactory bulb gains neurons at the same pace as the rest of brain (power exponent, 1.198 ± 0.252, which includes unity; *r*^2^ = 0.763, *p* = 0.0021; linear fit, *r*^2^ = 0.816, *p* = 0.0008; Figure 6A); and in primates and scandentia, the number of neurons in the olfactory bulb is not significantly correlated with numbers of neurons in the rest of brain (power function, *p* = 0.7902; Figure 6A). In contrast, the cerebral cortex gains neurons faster than the rest of brain in both glires and primates (exponents of 1.190 ± 0.110 and 1.723 ± 0.314, and *p* < 0.0001 and *p* = 0.0054, respectively; Figure 6B). The scaling relationships that apply across structures are summarized in Figure 7.

## Discussion

Here we find that the eulipotyphlan olfactory bulb gains neurons faster than the cerebral cortex across species, an unexpected finding in face of the decrease in the relative mass of the olfactory bulb and relative expansion of the cerebral cortex with increasing brain size. Moreover, in some eulipotyphlan species the olfactory bulb has as many or more neurons than the cerebral cortex, despite the larger size of the latter. These findings have several implications for mammalian brain evolution. First, the large and growing absolute numbers of neurons in the olfactory bulb of eulipotyphlans compared to the cerebral cortex dispute the notion that, while olfaction was emphasized in early mammals, other sensory systems necessarily became more important as cortex expanded in evolution (Rowe et al., 2011), such as vision in primates (Gilad et al., 2004). The fact that eulipotyphlans are now considered a later branch than afrotherians in mammalian evolution argues for a renewed importance of olfaction in the behavior of this group. Primates of all brain sizes do have many more neurons in the cerebral cortex than in the olfactory bulb, which is consistent with the expansion of other sensory systems, and eulipotyphlans have as many or more neurons in the olfactory bulb than in the cerebral cortex, which is consistent with a heavy reliance on olfaction. However, both groups have a similar range of numbers of neurons in the olfactory bulb. We note that the distribution of projection and intrinsic neurons, both excitatory and inhibitory, is different across structures such as the cerebral cortex, the olfactory bulb and the cerebellum, with presumably decreasing proportions of projection neurons across these three structures, respectively. However, given that both projection and intrinsic neurons are the basic units of information processing, combining the activity of thousands of synapses, we assume that total numbers of neurons are a valid proxy for total information processing capacity in each structure. Thus, if total numbers of neurons in the olfactory bulb can be considered an indication of the amount of olfactory processing that can be performed, then it must be conceded that the increasing reliance on other sensory systems such as vision in primates did not occur at the expense of olfaction, but in addition to it.

Second, our finding that the neuronal scaling rules that apply to the olfactory bulb appear to be shared by afrotherians and glires, but not eulipotyphlans or primates, concurs with the phylogenetic analysis that shows that modern eulipotyphlans are a more recent group than afrotherians and actually do not reflect the ancestral mammalian state. A parsimonious alternative is that modern glires and afrotherians (and possibly primates as well) retained the ancestral neuronal rules for building the olfactory bulb and for adding neurons faster to the cerebral cortex than to the olfactory bulb in glires and afrotherians, while eulipotyphlans evolved a new way of scaling the olfactory bulb, both with a higher density of neurons that allows for a larger number of neurons to be concentrated in a small volume, and with a higher rate of addition of neurons to the olfactory bulb than to the cerebral cortex.

The olfactory system is known to be malleable in evolution, depending on use. Activation of odorant receptor genes induces growth of the olfactory epithelium, which in turn induces turbinal growth and ossification (Rowe et al., 2011), and might be related to the support of larger numbers of neurons in the olfactory bulb and pyriform cortex. The olfactory bulb also gains neurons in adult life through the rostral migratory stream (Lois et al., 1996), and it is currently unknown whether an increased addition of neurons in adult life might contribute to the faster scaling of neurons in the olfactory bulb than in the cerebral cortex of eulipotyphlans. Odontocete cetaceans lack an olfactory bulb (Turner, 1891; Jacobs et al., 1971), and the structure is relatively very small in other aquatic mammals such as pinnipeds and the manatee (Reep et al., 2007), in line with the little use of chemical olfaction in the water by these particular mammalian species. Similarly, structures related to olfaction exhibit mosaic evolution in other groups, changing in size coordinately amongst themselves but in a manner that is unrelated to other brain structures (Barton and Harvey, 2000). The greater addition of neurons to the olfactory bulb than to the cerebral cortex in eulipotyphlans, in contrast to the opposite pattern in glires and the lack of a correlation in primates, implies that olfaction is an extremely important sense for eulipotyphlans, more so than for other mammals. This conclusion is further strengthened by recent behavioral studies. The eastern mole (*Scalopus aquaticus*) can navigate rapidly up odor gradients without using scent trails to localize prey. This is accomplished with serial sampling over time (sniffing and moving) combined with comparison of stereo nostril cues during each sniff (Catania, 2013). Both water shrews (*Sorex palustris*) and star-nosed moles (*Condylura cristata*) can sample odorants while submerged using underwater sniffing (Catania, 2006). This ability was thought impossible until these eulipotyphlans were filmed in slow-motion while foraging underwater. This remarkable underwater olfactory ability likely stems from behavior specializations and the mechanics of the naris, rather than hypertrophied olfactory structures *per se*. Thus, it is not inconsistent with lower densities of neurons in the olfactory bulb of star-nosed moles, which are clearly touch specialists (Catania and Kaas, 1997). In any case, there is little doubt olfaction plays a special role for eulipotyphlans.

Our data thus indicate the occurrence of mosaic evolution in the branching of eulipotyphlans from the common Laurasiatherian ancestor, with the olfactory bulb and cerebellum scaling differently from the common ancestor while the cerebral cortex and rest of brain retained the scaling rules also found in afrotherians and glires. Nevertheless, even though our results suggest that the afrotherian/glires scaling rules applied to the olfactory bulb (and also cerebral cortex; see Neves et al., 2014) of the ancestral eutherians, one should remain cautious when predicting the relative importance of olfaction in early mammals from sheer volume of brain structures, given that structure size does not predict number of neurons homogeneously across all mammals (Herculano-Houzel, 2011). It will be important now to look at the neuronal scaling rules that apply to the olfactory bulb of other branches of placental mammals, and to non-eutherian mammals: marsupials and monotremes. The prediction based on the current findings and hypothesis is that the cellular composition of the olfactory bulb of these latter groups should match those found for afrotherians and glires.

Here we show that, across species, the olfactory bulb gains neurons faster than the cerebral cortex in insectivores; at the same rate in glires; and independently in primates. This is in contrast to the olfactory bulb gaining mass at the same rate as the cerebral cortex across insectivore species and more slowly than the cerebral cortex across glires. Our finding that the relative scaling of numbers of neurons in the olfactory bulb compared to other brain structures differs from the scaling expected from mass relationships underscores the importance of not using volume indiscriminately as a proxy for numbers of neurons, which was common practice in the literature (for instance, Finlay and Darlington, 1995; Clark et al., 2001; Sultan, 2002).

One of the original uses of brain structure volumes to make inferences about mammalian brain evolution was by Stephan and Andy (1969), who put forward the notion that brain evolution equates with cortical expansion in detriment of other brain structures, and most of all olfactory bulb. Those authors used progression indices (akin to encephalization, calculated for each structure from the regression of their volume onto body mass) to show that neocortex is the structure that becomes relatively increased the most, while olfactory bulb is the only structure to “involute.” Using Stephan's data, Finlay later repeated this analysis and concluded that the olfactory bulb increases in volume faster than total brain size in insectivores and bats, but more slowly than total brain size in simians and prosimians (Finlay et al., 2001).

These conclusions contributed to the notion that, in mammalian evolution, olfaction became a less and less important sensory modality, especially in primates, given its relatively reduced “importance” as gauged from the relative volume of the olfactory bulb. This notion is disputed by our finding that the number of neurons in the olfactory bulb can be very large in eulipotyphlans, despite the relatively small size of the structure.

Importantly, our data also dispute the notion that primates are not as reliant on olfaction, a notion that has been based on the small relative size of the human olfactory bulb, compared to the brain (Baron et al., 1983), and recently supported by the finding that the loss of olfactory receptor genes coincides with the acquisition of full trichromatic vision in primates (Gilad et al., 2004). Those authors found that a twice higher percentage of olfactory receptor genes (32 against 16%) are pseudogenes in Old World monkeys and apes (which have full trichromatic vision) than in New World monkeys (which don't have full trichromatic vision). Although we were unable to examine the human olfactory bulb in this study, the neuronal scaling rules that apply to primates allow us to predict a total of 15–16 million neurons in the human olfactory bulb, given an average volume of 114 mm^3^ (which can be approximated to 114 mg; Baron et al., 1983). Surprisingly, this number of neurons falls within the same range as the number of neurons we found in the largest eulipotyphlan olfactory bulbs. Thus, while humans have been considered to be microsmatic based on the relative (not absolute) size of their olfactory bulb, they still have as many olfactory bulb neurons as are found to support olfaction in animals that rely heavily on it for their behavior and survival. This similarity is in line with the observation that the squirrel monkey has an unexpectedly high olfactory sensitivity (Laska et al., 2000), and with the more recent finding that human olfactory abilities are actually better than presumed (Porter et al., 2006). Gordon Shepherd challenged the belief that the behavioral importance of the sense of olfaction diminished during human evolution, and defended the notion that human olfaction serves food perception and seeking, as it is the main component of flavor (Shepherd, 2012). Thus, given the large absolute number of neurons predicted to compose their olfactory bulb, compared to macrosmatic eulipotyphlans, humans (and other primates; Laska et al., 2000) should no longer be considered microsmatic. Indeed, the absence of olfaction changes human life in ways that transcend flavor perception, for instance depriving individuals of an olfactory perception of their surrounding environment, which goes beyond simple object identification (Tafalla, 2013). We suggest that a decrease in the relative size of the olfactory bulb is to be expected in primates given the faster addition of neurons to their cerebral cortex than to other brain areas, but that does not imply a diminished olfactory capacity. We propose that a distinction be made between olfactory capabilities and relative reliance on olfaction. Eulipotyphlans have both large olfactory capabilities and rely heavily on them; primates still have strong olfactory capabilities, but rely more heavily on vision. These different strategies illustrate the principle of mosaic evolution in generating brain diversity: while expanding the cerebral cortex by adding neurons to it is expected to bring cognitive advantages, apparently eulipotyphlans do well enough with more neurons in the olfactory bulb than in the cerebral cortex.

Finally, we show that the absolute expansion of the cerebral cortex does not necessarily imply a relative expansion of the cerebral cortex in terms of the percentage of brain neurons concentrated in the cerebral cortex. This finding adds to the previous demonstration that, despite its relative expansion over the cerebellum across species, the cerebral cortex actually gains neurons at a similar rate as the cerebellum across species (Herculano-Houzel, 2010). Thus, although cortical expansion certainly is a prominent feature of brain evolution, it should no longer be equated with the notion of a cortical takeover at the expense of all other brain structures.

### Conflict of interest statement

The authors declare that the research was conducted in the absence of any commercial or financial relationships that could be construed as a potential conflict of interest.
